# Editorial: Recent applications of noninvasive physiological signals and artificial intelligence

**DOI:** 10.3389/fninf.2025.1543103

**Published:** 2025-01-16

**Authors:** Irma N. Angulo, Eduardo Iáñez, Andres Ubeda

**Affiliations:** ^1^Departamento de Ingeniería Biomédica, Escuela de Ciencias Aliadas de la Salud, Universidad de Monterrey, San Pedro Garza García, Mexico; ^2^Brain-Machine Interface Systems Lab, Engineering Research Institute (I3E), Miguel Hernández University, Elche, Spain; ^3^Human Robotics Group, University Institute of Physics Applied to Sciences and Technologies (IUFACyT), University of Alicante, Alicante, Spain

**Keywords:** biomedical signal processing, machine learning, brain-computer interface, affective computer, user experience

Artificial intelligence (AI) is currently transforming diverse fields (Qasmi and Fatima, 2024; Alyabroodi et al., 2023), by enabling the personalization of the user experience or expected outcomes, by monitoring and detecting pathological conditions, among other benefits. The use of non-invasive biomedical signals can enhance the performance of AI applications by providing complimentary objective information about the traits of a person that can be otherwise difficult to evaluate or by delivering physiological information that can contribute to the advancement of biomedical signal processing to improve medical attention (Tseng et al., 2023; Kumar et al., 2024).

Several applications of AI on non-invasive physiological signals for neuroscience are explored in this collection. In the pioneer field of neuro-humanities, Blanco-Ríos et al. propose a real-time system for emotion recognition using EEG signals and Extra-Trees, which achieved a very high accuracy with the goal of enhancing learning experience in the field of humanities. Health applications are also explored, such as in Fernandez Rojas et al. where the accuracy of deep learning models is compared with functional near-infrared spectroscopy (fNIRS) and baseline models for the assessment of pain. The best model (CNN-LSTM) could be used as a possible method for effective pain assessment, which could lead to a more precise tool for clinicians for the care for patients with communication limitations.

Other novel studies in this collection are focused on the research of brain-computer interfaces (BCI). Juan et al. combined spectro-temporal and spectro-spatial feature extraction methods and deep learning models (CNN) to enhance the decoding accuracy of motor imagery from EEG signals during pedaling tasks, obtaining an accuracy of up to 80% despite higher instability. In a similar topic, Dillen et al. evaluated the usability of a BCI that relied on motor imagery detection and augmented reality for different motor tasks. This was achieved through an assessing protocol that consisted in validating technical robustness, evaluating the control system and comparing it with a non-BCI alternative, including user evaluations. These contributions are important for assuring BCIs are practical and effective in different scenarios.

Regarding wearable devices, biomedical signals need to be compressed and reconstructed for their transmission while reducing noise. Zhang et al. present an improvement in electrocardiographic (ECG) signal reconstruction based on weighted nuclear norm minimization (WNNM) and denoising-based approximate message passing algorithms (AMP). Within the same topic of signal quality, Cisotto et al. present an innovative deep learning model called hvEEGNet, which is based on a hierarchical variational autoencoder and trained with a new loss function. It is designed for the reconstruction of multi-channel EEG signals. Unlike previous works, the model is capable of performing high-fidelity reconstruction of multi-channel EEG datasets, with high consistency across subjects.

The presented articles in this Research Topic provide insights into the recent applications of artificial intelligence and biomedical signals, mainly with a neuroscience perspective. These studies show some of the future trends that may be developed to improve personal experience and accuracy, especially in the healthcare services.
